# In Vitro Profiling of the Antiviral Peptide TAT-I24

**DOI:** 10.3390/ijms251910463

**Published:** 2024-09-28

**Authors:** Theodhora Ziu, Ezgi Sambur, Zsolt Ruzsics, Hartmut Hengel, Reingard Grabherr, Siegfried Höfinger, Hanna Harant

**Affiliations:** 1 Pivaris BioScience GmbH, Media Quarter Marx 3.4, Maria-Jacobi-Gasse 1, 1030 Vienna, Austria; office@pivaris-bioscience.at; 2 VSC Research Center, Technical University of Vienna, Operngasse 11/E057-09, 1040 Vienna, Austria; ezgisambur@gmail.com (E.S.); or shoefing@mtu.edu (S.H.); 3 Institute of Virology, Medical Center and Faculty of Medicine, University of Freiburg, Hermann-Herder-Str.11, 79104 Freiburg, Germany; zsolt.ruzsics@uniklinik-freiburg.de (Z.R.); hartmut.hengel@uniklinik-freiburg.de (H.H.); 4 Institute of Molecular Biotechnology, Department of Biotechnology, University of Natural Resources and Life Sciences Vienna, Muthgasse 18, 1190 Vienna, Austria; reingard.grabherr@boku.ac.at; 5 Department of Physics, Michigan Technological University, Houghton, MI 49931, USA

**Keywords:** antiviral peptide, cell-penetrating peptide, peptide variants, baculovirus, mouse cytomegalovirus, molecular modeling

## Abstract

The synthetic peptide TAT-I24 (GRKKRRQRRRPPQCLAFYACFC) exerts antiviral activity against several double-stranded (ds) DNA viruses, including herpes simplex viruses, cytomegalovirus, some adenoviruses, vaccinia virus and SV40 polyomavirus. In the present study, in vitro profiling of this peptide was performed with the aim of characterizing and improving its properties for further development. As TAT-I24 contains three free cysteine residues, a potential disadvantageous feature, peptide variants with replacements or deletions of specific residues were generated and tested in various cell systems and by biochemical analyses. Some cysteine replacements had no impact on the antiviral activity, such as the deletion of cysteine 14, which also showed improved biochemical properties, while the cyclization of cysteines 14 and 20 had the most detrimental effect on antiviral activity. At concentrations below 20 µM, TAT-I24 and selected variants did not induce hemolysis in red blood cells (RBCs) nor modulated lipopolysaccharide (LPS)-induced release of cytokines, such as interleukin-6 (IL-6) and tumor necrosis factor-α (TNF-α), in human peripheral blood mononuclear cells (PBMCs). These data indicate that TAT-I24 or its peptide variants are not expected to cause unwanted effects on blood cells.

## 1. Introduction

For the prevention or treatment of viral infections, various medications are available but mostly limited to specific types of viruses [1,2,3]. Considering the emergence of new viruses due to global changes in climate and wildlife populations, there is a high unmet medical need to develop novel, broad-acting therapeutics [4,5]. This need was dramatically highlighted by the SARS-CoV-2 pandemic, with millions of deaths and a long-lasting socio-economic impact [6]. Apart from small molecules, antimicrobials, in particular antiviral peptides, are currently in development [7,8,9,10]. Numerous peptides have been identified that exert antiviral activity and that were either identified from natural sources, such as peptides in venoms from various species [11], or produced endogenously, such as defensins [12,13,14,15,16,17]. Other antiviral peptides include synthetic peptides, such as fusion inhibitors, which are derived from proteins involved in viral membrane fusion [18,19,20,21,22,23]. Peptides can also target endosomal acidification, such as P9R [24], or viral membrane integrity, such as LL-37 [25,26]. 

Peptides can have several advantages over small molecules, such as their target specificity and interference with protein–protein interactions due to their larger sizes. Although small molecules have been used for decades in antiviral treatments, major issues are toxicities and resistance formation in long-term treatments [1,2,3]. However, the possible disadvantages of peptides are the lack of cell penetration, instability due to degradation by proteases, as well as lower potencies [8,9]. Some of these features can be improved by either modification of peptides, such as by PEGylation, a conjugation to polyethylene glycol, or by use of D-amino acid residues and fusion to cell-penetrating peptides [27,28]. Despite these difficulties, antiviral peptides have already entered the market, such as the fusion inhibitors Fuzeon^®^ (enfuvirtide) for the treatment of HIV-1 infections [29] and Hepcludex^®^ (bulevirtide) for the treatment of chronic hepatitis D infections [30]. 

We have identified the antiviral peptide TAT-I24, which has broad-acting activity against several double-stranded (ds) DNA viruses in vitro [31,32]. For the further development of this peptide, it is necessary to investigate its properties and how the peptide can be modified while retaining its antiviral activity. As TAT-I24 contains three free cysteine residues, a generally undesired property for further development, individual cysteine residues were replaced, and the impact of these modifications on antiviral activity was investigated. Furthermore, the stability of TAT-I24 as well as the effect of TAT-I24 and selected variants on immune cells were studied. 

## 2. Results and Discussion

### 2.1. Effect of Cysteine Replacements on Antiviral Activity of TAT-I24

TAT-I24 is a synthetic, linear peptide consisting of the cell-penetrating TAT-peptide (amino-acid residues 48–60) [33,34,35] fused to the 9-mer peptide I24 with the sequence CLAFYACFC [31]. Originally, the peptide I24 was found to inhibit reporter gene expression when transfected with plasmid DNA into cells through the binding of I24 to the plasmid DNA [31]. The TAT peptide itself has also been shown to bind DNA mainly due to unspecific electrostatic interactions [36,37]. However, DNA binding was greatly enhanced by fusion with the I24 peptide, as in TAT-I24 [32]. Due to their positive net charge, cationic peptides also bind components on the cell surface, such as heparan sulfate proteoglycans [38,39,40], which are also used by several viruses, in addition to specific receptors, for cell attachment [41,42,43,44]. These combined features led to a peptide with antiviral activity against several dsDNA viruses, exemplified by its effect on transgene expression after the transduction of mammalian cells with a baculovirus vector. The antiviral effect of TAT-I24 was further confirmed with several dsDNA-viruses, such as herpes simplex viruses, cytomegalovirus, some but not all human adenoviruses, SV40 polyomavirus and vaccinia virus [31,32].

The biochemical properties of TAT-I24 align with those of several antiviral peptides, which frequently have amphipathic structures containing basic and hydrophobic amino acid residues [8]. Indeed, using the FIRM-AVP algorithm [45], TAT-I24 was highly scored as an antiviral peptide (score = 0.94). For the consideration of this peptide as a potential antiviral therapeutic or for the prophylactic treatment of infections with dsDNA viruses, a systemic application would be required. However, the development of therapeutic peptides for systemic applications is still a major hurdle. While peptides have several advantages, such as excellent target specificities and low toxicities [46], aggregation and oligomerization [47,48], poor serum stability [49,50] and interaction with other factors are challenges in the development of peptide therapeutics [51]. One particular critical feature of TAT-I24 is the presence of three cysteines. Free sulfhydryl (SH) groups are usually undesired, as they are highly reactive and can cause oligomerization, aggregation and unwanted absorption due to protein binding [52]. On the other hand, free cysteines can also stabilize a peptide through binding to the free cysteine 34 in albumin [53]. Thiol-containing peptides were also shown to interact with free thiols on the cell surface and facilitate the binding and internalization of the peptide [54]. The addition of a single cysteine residue to the TAT peptide has been reported to exert antiviral activity [55], and a single cysteine residue in the cell-penetrating peptide AP fused to an enhanced green fluorescent protein (GFP) has been shown to be essential for cellular uptake [56]. The cell-penetrating peptide CyLop-1 contains three cysteines and has been reported to be an effective agent for the cytosolic delivery of cargo [57]. It is, therefore, likely that the three cysteines in TAT-I24 play an important role in cell attachment and its antiviral effect. To further test this hypothesis, peptide variants with replacements of individual cysteines within the TAT-I24 sequence were synthesized, aiming at improving its physico-chemical properties while, at the same time, retaining full antiviral activity. The consequences of individual replacements on the antiviral effect of the peptides were analyzed by employing two established in vitro models of dsDNA viruses: first, the transduction of HEK293 cells with a non-replicating baculovirus vector, and second, reporter gene expression upon infection of permissive NIH/3T3 cells with replication-competent recombinant mouse cytomegalovirus (MCMV) [31,58]. In addition, all peptides were further tested for potential cytotoxicity using HEK293 cells and analyzed for their capacity to bind dsDNA [32].

Baculovirus-mediated firefly luciferase transgene expression, when transduced into HEK293 cells, was highly sensitive to inhibition by TAT-I24 at low peptide concentrations, while the inhibition of MCMV-mediated luciferase expression in fibroblasts required some higher concentrations of TAT-I24 (Table 1). Also, baculovirus-mediated reporter gene expression was less sensitive to amino acid changes in the individual peptide variants as compared to MCMV-infected NIH/3T3 cells, where specific amino acid substitutions more severely affected virus-mediated luciferase expression. Accordingly, both systems were analyzed in parallel, revealing comparable trends in relative sensitivities (Table 1). 

**Figure 1:**
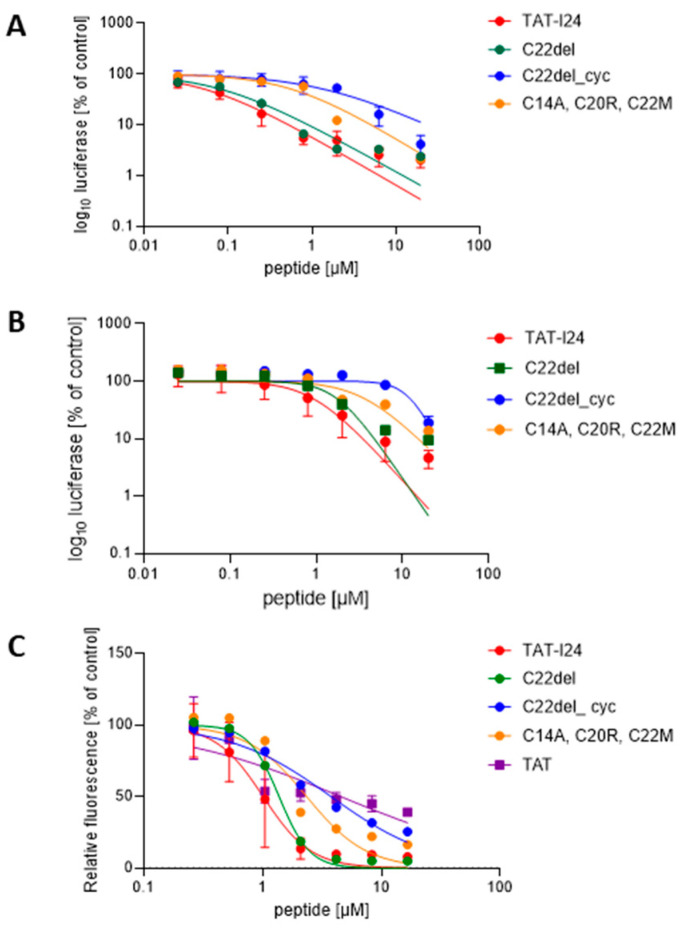
Sensitivities to selected TAT-I24 peptide variants in two infection models and dsDNA binding of variants. (**A**) luciferase reporter gene expression after transduction of HEK293 cells with baculovirus, performed once as duplicate and twice as triplicate analyses (*n* = 8). (**B**) luciferase reporter gene expression in NIH/3T3 cells after infection with replication-competent MCMV, performed in three triplicate analyses (*n* = 9). (**C**) dose-dependent DNA binding of selected peptides, performed once as duplicate and twice as triplicate analyses, *n* = 8. Curve fitting was performed using a non-linear regression model.

Replacements of cysteine at position 14 (C14) by glycine or the aliphatic amino acids alanine or leucine only marginally reduced the inhibiting effect on baculovirus- or MCMV-mediated reporter gene expression. Even the deletion of C14 did not impair its antiviral activity. However, the introduction of a proline residue at position 14 instead of a cysteine caused some reduction in antiviral activity against MCMV, indicating a potential relevance of peptide conformation for antiviral activity. Introducing an arginine residue at position 14 also led to some reduction in the antiviral effect against MCMV. Single replacements of cysteine 22 by serine (C22S) or glycine (C22G) did not severely affect its antiviral activity. However, the deletion of C22 (C22del) and the additional replacement of C20 with arginine (C20R, C22del) caused a slight reduction in antiviral activity compared to TAT-I24 in MCMV-infected cells. The replacement of all three cysteines—C14 by alanine, C20 by arginine and C22 by methionine (C14A, C20R, C22M)—caused further reductions in antiviral effects in both infection models but still had some residual antiviral activity at higher concentrations (Table 1, Figure 1A,B). This indicates that the cysteines in TAT-I24 positively contribute to the antiviral effect but can be reduced depending on the sensitivity of the infection model. 

The change with the highest impact on the antiviral activity of TAT-I24 was observed when C22 was deleted and a cyclization was introduced between C14 and C20 (C22del_cyc). In contrast to the linear peptide C22del, cyclization led to a strong reduction of the antiviral effect, indicating that conformation and free SH-groups are essential for the antiviral effect (Table 1, Figure 1A,B). 

DNA-binding of the peptides was analyzed using an assay based on the reduction of the fluorescence of DNA-bound SYBR^TM^ Gold dye in the presence of peptide. As a reference, the TAT peptide was included, which also binds DNA and causes a decrease in fluorescence but is less pronounced compared to TAT-I24 [32]. The cyclized peptide C22del_cyc, which had strongly reduced antiviral activity, also exhibited clearly reduced DNA binding (Figure 1C). A slight reduction in DNA binding was also seen for the variants C14A, C14L, C14G and C14P, as well as for the variant C20R, C22del and the variant C14A, C20R, C22M, demonstrating that DNA binding is important for its antiviral activity but not sufficient, thus indicating that additional mechanisms are involved (Table 1, Figure 1C). Cell viability data were obtained for all variants and showed that CC_50_ (the concentration of cytotoxicity 50%) was >20 µM in all cases (Supplementary Figure S1). The selectivity index (SI), that is, the ratio between CC_50_ and IC_50_, was calculated for all peptides tested in HEK293 cells, except for C22del and C22del_cyc, for which the CC_50_ could not be calculated from the results of the cell viability assay (Table 1, Supplementary Figure S1).

### 2.2. Computational Biology Support

Building on previous dsDNA affinity estimates based on the NMR structure 6MCE [32,59], the idea was to extend such computational considerations and investigate the consequences of substituted cysteine residues on the simulated binding strength (the binding of TAT-I24 variants to dsDNA formed by a 22-mer helical hairpin made of GC base pairs). If performed independently for residues Cys14, Cys20 and Cys22, a rather complete picture should arise that ideally matches the biological assays and potentially complements the list of examined TAT-I24 variants with non-intuitive candidates. Given the large dimension of TAT-I24 in relation to its binding target, i.e., a domain of dsDNA of comparable size, the first question was to address the structural stability of such complexes and the corresponding reproducibility of binding free energy calculations. Supplementary Figure S2 summarizes a 3-fold replication of the basic computation with a detailed analysis of complex-forming interactions, the degree of the structural integrity of the dsDNA and associated estimates of ∆_Gcmplx_. Variability turns out to be sizeable; hence, the resulting predictions should be considered as estimates only. Detailed structural snapshots of relevant complex geometries are given in Supplementary Figure S3, where the locations of cysteine residues are indicated in green. The resulting affinity calculations of all cysteine substitutions (three replicates) are summarized in Supplementary Table S1. An empirical requirement considered here is to only take into account results with variances smaller than 1000. The top 3 candidates for experimental verification are C14K, C14A and C14I for cysteine 14; C20R, C20E and C20I for cysteine 20; and C22N, C22R, C22M and C22K for the C-terminal cysteine 22 (see the green cells in Supplementary Table S1). Selected peptide candidates with substitutions following these suggestions were synthesized and included in the test list, for example, in peptide C14A, the replacement of C20 by arginine as in peptide C22del, C20R and in peptide C14A, C20R, C22M, which displays the simultaneous replacement of all three cysteines (Table 1). However, although plain DNA binding is a key parameter in the antiviral activity of this peptide, its antiviral action may still depend on additional features of the peptide, such as cell surface binding and its effect on virus entry, as some viruses are not sensitive to TAT-I24 [31].

### 2.3. Effects of Peptide Variants on Early Stages of Virus Infection

**Figure 2:**
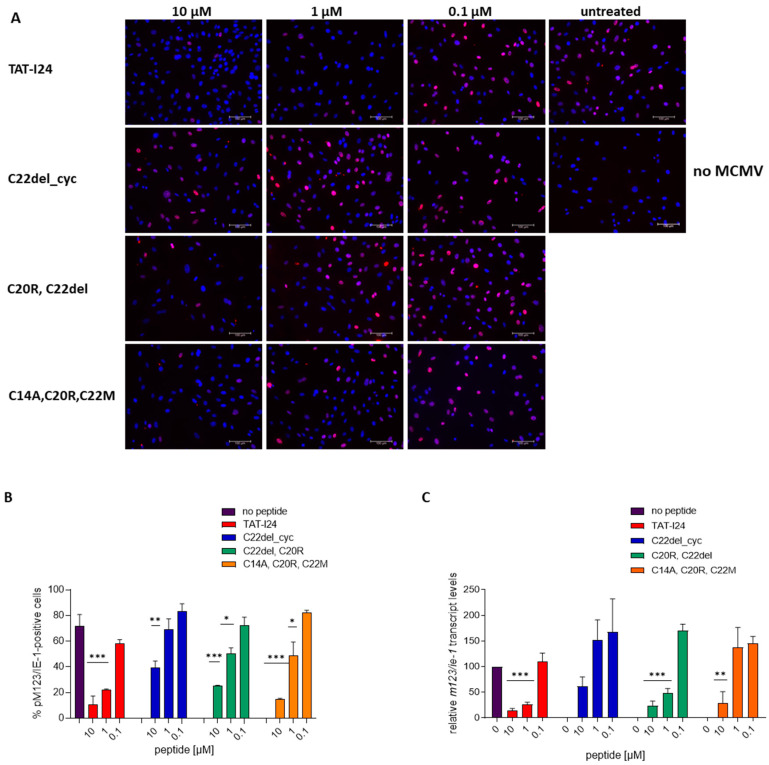
Effect of peptides on pM123/IE1 protein and *m123*/*ie-1* gene expression. (**A**) Representative microscopic images (20× objective) of pM123/IE1 protein (red fluorescence) in MCMV-infected NIH/3T3 cells 6 h post-infection in the presence of three concentrations of peptides. Nuclei are stained with DAPI (blue fluorescence). Scale bars indicate 100 µm. (**B**) Percentage of pM123/IE1-positive cells in MCMV-infected NIH/3T3 cells in the presence of peptides. Mean ± SD from two images is shown. (**C**) Relative *m123/ie-1* transcript levels (% of MCMV-infected control) two hours post-infection. Mean ± SD from duplicate treatments is shown. Multiple *t*-test was used for statistical analysis; * represents statistically significant at *p* ≤ 0.05; ** represents statistically significant at *p* ≤ 0.01; *** represents statistically significant at *p* ≤ 0.001.

The antiviral effects of the peptides were determined by the extent of luciferase reporter gene expression after 24 h (baculovirus vector transduction) and 72 h (MCMV replication) post-infection. It has been shown earlier that TAT-I24 already acts at very early stages of virus infection [31,32]. It was, therefore, examined whether the differences in the potencies of the peptide variants could also be observed at earlier time points where viral gene expression starts. The activation of the MCMV immediate early *ie1*/*ie3* transcription unit is controlled by the major immediate early promoter/enhancer (MIEP) and requires extensive differential splicing [60]. The expression of the *ie1* gene of MCMV is reduced by TAT-I24 at the transcript level [61]. To analyze the protein expression of pM123/IE1, NIH/3T3 cells were infected with MCMV in the absence or presence of 10-fold dilutions of peptides, and pM123/IE1 expression was analyzed 6 h post-infection by immunofluorescence [62]. While after infection with MCMV, a high percentage of cells stained positive for pM123/IE1, the number of positive cells was clearly reduced when infection was performed in the presence of 1 and 10 µM TAT-I24. Two less active variants, peptide C20R, C22del and peptide C22del_cyc, showed a higher number of pM123/IE1-positive cells, as compared to TAT-I24-treated cells at 1 µM, but also resulted in a reduced number of pM123/IE1-positive cells at a concentration of 10 µM, thus confirming the partial response observed with their effect on MCMV replication. The same applied to the variant C14A, C20R, C22M, which was still able to reduce the number of pM123/IE1-positive cells when applied at 10 µM (Figure 2A,B). These results were also reflected at the level of *m123*/*ie-1* transcripts. Cells were treated with the peptides at three different concentrations and infected with MCMV. Two hours post-infection, RNA was isolated and *m123*/*ie-1* transcript levels were analyzed by qPCR relative to infected cells without peptides [61]. While TAT-I24 and C20R, C22del significantly reduced *m123*/*ie-1* transcript levels at concentrations of 1 and 10 µM, variant C14A, C20R, C22M only significantly reduced *m123*/*ie-1* transcript levels at 10 µM (Figure 2C). The peptide C22del_cyc, which was the least active in reducing the number of pM123/IE-1-positive cells, also did not significantly reduce *m123*/*ie-1* transcript levels. Overall, the results from the pM123/IE-1 staining are also reflected at the level of *m123*/*ie-1* RNA (Figure 2C). 

**Figure 3:**
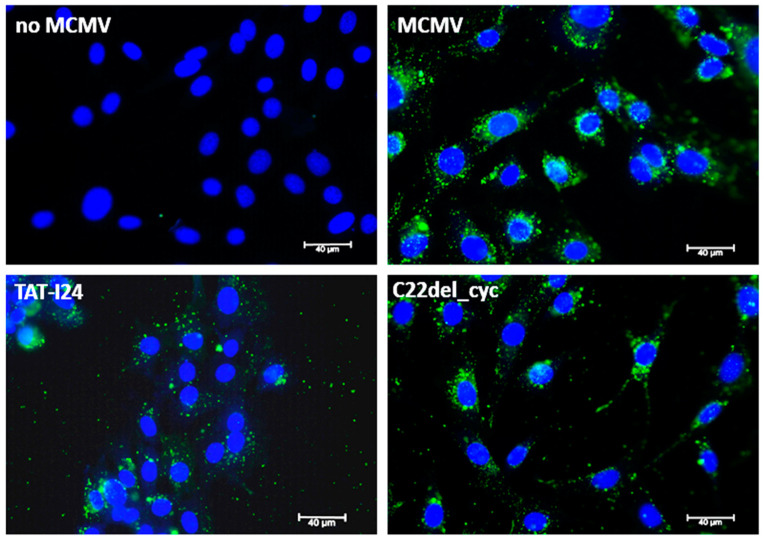
Early effects of peptides during MCMV entry. Microscopic images from cells, non-infected or infected with a DiO-labeled MCMV (green fluorescence) for 120 min in the absence or presence of 10 µM peptides (40× objective). Nuclei are stained with DAPI (blue fluorescence). Scale bars indicate 40 µm.

To further investigate events at early stages upon virus entry, MCMV was directly labeled with Vybrant™ DiO Cell-Labeling Solution. This dye and others, such as DiD, belong to a group of lipophilic long-chain dialkylcarbocyanines, which can stain membranes and are used for single-virus tracking [63,64,65]. In cells infected with DiO-labeled MCMV in the absence of peptides, fluorescent foci resembling viral membranes in the cytosol and surrounding the nuclei were observed 120 min post-infection (Figure 3). However, in the presence of TAT-I24, the fluorescent signal appeared with less perinuclear organization and was more dispersed in the whole cytoplasm, whereas in the presence of the less active peptide variant C22del_cyc, the organization of the fluorescent particles more closely resembled the organization seen in cells that were infected without peptide (Figure 3). 

### 2.4. Effect of TAT-I24 and Its Variants on Blood Cells 

Antimicrobial peptides can disrupt cell membranes or cause toxic effects within cells [63,66]. It was, therefore, examined whether the antiviral effect of TAT-I24 and its variants could be linked to their interaction with membranes. Indeed, morphological changes, such as cell aggregation, were observed at higher concentrations of TAT-I24 (>10 µM) but were not associated with cell toxicity [31]. 

**Figure 4:**
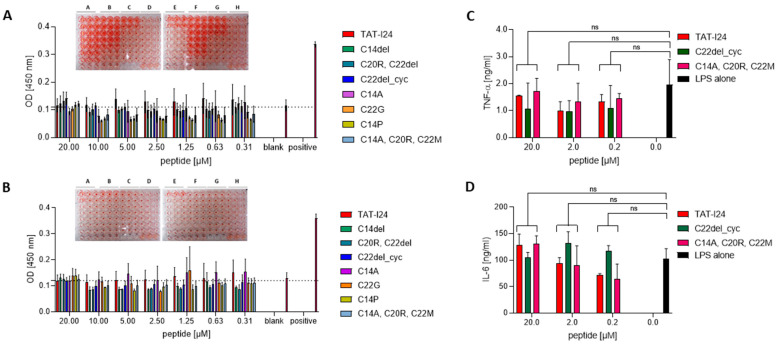
TAT-I24 and peptide variants neither induce hemolysis nor cause immunomodulatory effects. OD [450 nm] is shown from supernatants after incubation of RBCs with peptide dilutions prepared in PBS (**A**) or PBS + 10% donor serum (**B**). Experiments were performed twice in triplicate (*n* = 6). Insets show the plates after incubation and centrifugation of RBCs with peptides (A = TAT-I24; B = C14del; C = C20R, C22del; D = C22del_cyc; E = C14A; F = C22G; G = C14P; H = C14A, C20R, C22M). TAT-I24 and peptide variants do not significantly alter TNF-α (**C**) and IL-6 levels (**D**) in LPS-stimulated PBMCs. Results shown are as mean ± SD from each peptide treatment performed in duplicate. Multiple *t*-test was used for statistical analysis (ns—not significant; *p* > 0.05).

A selection of eight peptides with different antiviral activity was, therefore, tested for their potential to induce hemolysis in human red blood cells (RBCs) [63]. Peptide dilutions were either prepared in phosphate-buffered saline (PBS) or in PBS supplemented with 10% donor serum. Plates with peptide dilutions prepared in PBS showed that, after incubation and centrifugation, RBCs exhibited a larger, clearly visible pellet, an effect that is associated with the swelling of cells (inset in Figure 4A and Supplementary Figure S4). However, no signs of hemolysis could be observed at the concentrations used (Figure 4A). When peptides were diluted in the presence of donor serum, neither RBC swelling nor hemolysis was observed, indicating that the interaction of peptides with serum components reduces their effect on membranes (inset in Figure 4B). Those peptides, which were least active in the virus infection models, also caused reduced swelling of RBC, such as variant C20R, C22del, peptide C22del_cyc; variant C14P; and variant C14A, C20R, C22M (Figure 4A). Of note, variant C14A, which had full antiviral activity, caused less RBC swelling compared to TAT-I24 (inset Figure 4A). 

Several short antiviral peptides have been reported to exert immunomodulatory effects by either enhancing or reducing levels of inflammatory cytokines, such as IL-6, IL-1β and TNF-α [67,68,69]. To address a potential immunomodulatory effect of TAT-I24 and some of its variants, human PBMCs were isolated from healthy volunteer donors and treated with three concentrations of TAT-I24 and the less active peptide C14A, C20R, C22M or the variant C22del_cyc and stimulated with LPS for 24 h. Supernatants were then collected, and the levels of IL-6 and TNF-α were quantified by ELISA. No significant differences in LPS-induced IL-6 or TNF-α release between peptide-treated PBMCs and the control without peptides were observed (Figure 4C,D). Together, TAT-I24 and its variants are not expected to cause effects on pro-inflammatory cytokine levels.

### 2.5. Biochemical Analysis of TAT-I24 and Variant C14del

**Figure 5:**
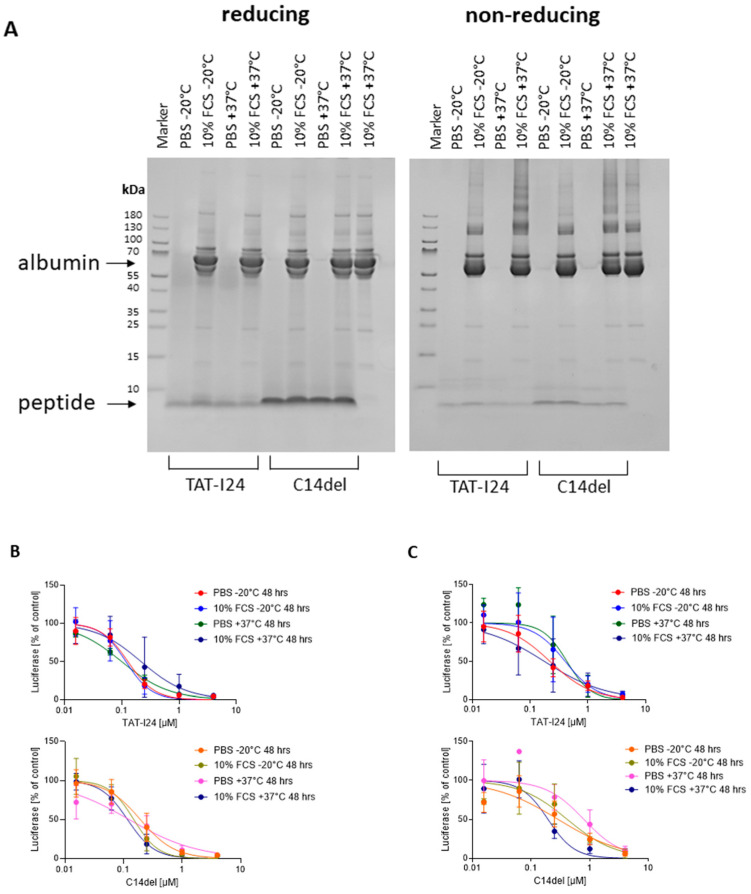
Stability of TAT-I24 and C14del in PBS or PBS with 10% FCS. (**A**) Coomassie stain of peptides separated by SDS-PAGE under reducing conditions (**left**) or non-reducing conditions (**right**). (**B**) Antiviral effect of peptides after incubation for 48 h at −20 °C or +37 °C determined in HEK293 cells transduced with baculovirus 24 h post-infection and (**C**) in NIH/3T3 cells infected with MCMV 72 h post-infection. Results shown are mean ± SD from two independent peptide incubations performed in duplicate analyses.

To address potential oligomerization and aggregation, as well as the stability of the peptides in serum, TAT-I24 and the peptide C14del were incubated at 37 °C for 48 h in the presence of PBS or PBS containing 10% fetal calf serum (FCS). The peptides were then applied to SDS–polyacrylamide gel electrophoresis (SDS-PAGE), followed by Coomassie Brilliant Blue staining. Under reducing conditions, where disulfide bonds are disrupted, a band of the TAT-I24 monomer was detected at the expected size in all samples and an additional weak higher molecular weight smear around 45 kDa, indicating possible oligomer formation, which could not be resolved by the treatment of the samples (Figure 5A, left). However, the peptide C14del showed a much clearer staining of the monomeric peptide under these conditions (Figure 5A, left). When the peptides were heated with an SDS loading buffer but without a reducing agent, only faint bands of monomers were observed with both peptides, indicating oligomer formation under non-reducing conditions, independent of the presence of FCS (Figure 5A, right). 

In addition, antiviral activity was determined by the baculovirus transduction assay with HEK293 cells (Figure 5B) and in MCMV-infected NIH/3T3 cells (Figure 5C). A 48 h incubation of TAT-I24 in the presence of 10% FCS led to a slight reduction in antiviral activity, indicating a possible binding to serum components, such as albumin, as reported for other antimicrobial peptides [70]. After incubation of the peptide at 37 °C in the absence of the serum, curve shaping for both peptides was altered (Figure 5B,C). In contrast to TAT-I24, the peptide C14del was more stable in the presence of serum, indicating that the properties of the peptides could be improved upon the replacement of specific cysteine residues.

## 3. Materials and Methods

### 3.1. Peptides

TAT-I24 was synthesized at Bachem AG (Bubendorf, Switzerland). All other peptides were synthesized at Synpeptide Co., Ltd. (Shanghai, China), with a purity > 95%. The peptide C14A, C20R, C22M was synthesized at ProteoGenix (Schiltigheim, France). All peptides were dissolved in dimethyl sulfoxide (Sigma Aldrich, Schnelldorf, Germany) at a concentration of 10 mM and stored at −20 °C. For all peptide treatments in cell culture experiments, the maximal DMSO concentration was 0.2%, a concentration where no toxic effects on the cells were seen in our previous studies [31,32].

### 3.2. Cell Culture

HEK293 and NIH/3T3 cells were grown in a CO_2_-independent medium supplemented with 10% FCS, 2 mM glutamine and 1% antibiotic–antimycotic (ThermoFisher, Darmstadt, Germany) and cultivated in a humidified atmosphere at 37 °C. Cells were passaged once a week. 

### 3.3. Baculovirus

A baculovirus expressing luciferase under the control of the CMV promoter has been described previously [31]. HEK293 cells were seeded at a density of 2 × 10^4^ cells/100 µL per well in 96-well plates (Sarstedt, Germany). On the next day, cells were treated with a 1:3 dilution series of peptides and, without further pre-incubation, subsequently infected with baculovirus–luc at a multiplicity of infection (MOI) of 5. Cells were lysed 24 h post-infection using 20 µL Cell Culture Lysis Reagent (Promega, Mannheim, Germany). From the lysates, 10 µL was transferred to white 96-well plates, and luciferase was recorded using a luciferase assay system and a GloMax^®^-Multi Detection System (Promega, Mannheim, Germany).

### 3.4. Mouse Cytomegalovirus

NIH/3T3 cells were seeded at a density of 2 × 10^4^ cells/100 µL per well in a 96-well plate (Sarstedt, Germany) and allowed to attach overnight. On the next day, cells were treated with a 1:3 dilutions series of peptides and, without further pre-incubation, subsequently infected with the MCMV strain delm157-luc rep [58]. The MCMV stock was prepared and quantified as previously described [31]. The virus (MOI 0.5 for luc assays; for other assays, see the specific descriptions) was adsorbed to cells with the aid of centrifugal enhancement twice at 800× *g* for 15 min at room temperature and incubated at 37 °C for the indicated times. For the luciferase assays, the infected cells were lysed 72 h post-infection using 20 µL Cell Culture Lysis Reagent (Promega, Mannheim, Germany). From the lysates, 10 µL was transferred to white 96-well plates, and luciferase was recorded using a Luciferase Assay System and a GloMax^®^-Multi Detection System (Promega, Mannheim, Germany).

### 3.5. Staining and Microscopy

NIH/3T3 cells were seeded at a density of 2 × 10^4^ cells/250 µL per well in ibiTreat 8-well chambers (ibidi, Gräfelfing, Germany) and allowed to attach overnight at 37 °C. On the next day, cells were treated with peptides and infected with MCMV at an MOI of 1 with the aid of centrifugal enhancement, twice at 800× *g* for 15 min. After 6 h of incubation at 37 °C, the supernatants were removed and cells were fixed with 5% formaldehyde for 1 h at room temperature, followed by permeabilization with 0.5% Triton X-100 (Sigma Aldrich, Schnelldorf, Germany) in PBS for 10 min at room temperature. Cells were then incubated with 3% bovine serum albumin (BSA; Sigma Aldrich, Schnelldorf, Germany) in PBS for one hour. After that, anti-m123/IE-1 antibody (clone IE1.01; Center for Proteomics, Rijeka, Croatia) was added at a 1:400 dilution in PBS + 1% BSA and slides were incubated overnight at 4 °C. On the next day, wells were washed twice with PBS. The secondary antibody, goat anti-mouse IgG Texas Red (#T6390; (ThermoFisher, Darmstadt, Germany), diluted 1:100 in PBS + 1% BSA, was added and slides were incubated for one hour at room temperature. After two washes with PBS, 115 µL of a solution of 1 µg/mL 4′,6-Diamidino-2-Phenylindole, Dihydrochloride (DAPI; Invitrogen/ThermoFisher, Waltham, MA, USA) was pipetted in each well and wells were incubated at room temperature for 15 min. DAPI solution was then replaced by PBS, and slides were inspected by microscopy with a 20× objective. 

DiO labeling was performed by incubation of MCMV (2 × 10^7^ pfu) with 5 µL of Vybrant™ DiO Cell-Labeling Solution in 300 µL cell culture medium supplemented with 10% FCS for 20 min at 37 °C. After that, the virus was precipitated overnight at 4 °C by addition of 600 µL autoclaved solution of polyethylene glycol/sodium chloride (30% (*w*/*v*) PEG-8000 (Sigma Aldrich, Schnelldorf, Germany), 10.5% NaCl (*w*/*v*) in PBS, followed by centrifugation on the next day for one hour at 1600× *g* at 4 °C. Supernatants were discarded, and the precipitated virus was resuspended in 500 µL cell culture medium supplemented with 10% FCS, aliquoted and stored at −80 °C. 

NIH/3T3 cells were seeded at a density of 2 × 10^4^ cells/250 µL per well in ibiTreat 8-well chambers (ibidi, Germany) and allowed to attach overnight at 37 °C. On the next day, cells were treated with peptides and infected with DiO-labeled MCMV corresponding to an MOI of approximately 5 with the aid of centrifugal enhancement, twice at 800× *g* for 15 min. After 2 h of incubation, cells were washed twice with PBS and fixed with 5% formaldehyde for 1 h at room temperature. Then, cells were stained with DAPI for 15 min, as described above. DAPI solution was then replaced by PBS, and slides were inspected by microscopy with a 40× oil objective using a Live Cell Video Microscope (Leica Microsystems, Wetzlar, Germany) provided by the BOKU Imaging Center (Vienna, Austria). 

### 3.6. RNA Isolation, cDNA Synthesis and Quantitative Real-Time PCR

NIH/3T3 cells were seeded at a density of 2 × 10^4^ cells/250 µL per well in a 48-well plate and allowed to attach overnight at 37 °C. On the next day, cells were treated with peptides at the indicated concentrations and infected with MCMV at an MOI of 1 with the aid of centrifugal enhancement, twice at 800× *g* for 15 min. Cells were then returned to 37 °C for two hours before cell lysis for further processing. RNA isolation, DNase digestion, cDNA reaction and qPCR were performed, as described previously [61].

### 3.7. Cell Viability Assay

HEK293 cells were seeded at a density of 2.5 × 10^3^ cells/100 µL per well in a 96-well plate (Sarstedt, Germany). In addition, a serial 1:2 dilution of the cell suspension was added to each plate to generate a standard curve. On the next day, cells were treated with serial 1:3 peptide dilutions in triplicate and incubated for 72 h at 37 °C. Then, 20 µL of CellTiter-Blue^®^ Cell Viability Assay (Promega, Mannheim, Germany) was added to each well and incubated at 37 °C for a further 1–2 h. Fluorescence was recorded using a GloMax^®^-Multi Detection System (Promega, Mannheim, Germany). The percentage of viable cells was calculated from the cell dilution series from each plate by linear regression analysis. The selectivity index was calculated using the following formula: SI = CC_50_/IC_50_.

### 3.8. DNA Binding Assay

DNA binding was detected using an assay based on reduction of fluorescence emitted by SYBR^TM^ Gold (ThermoFisher, Darmstadt, Germany) bound to dsDNA in the presence of peptides using the pcDNA3.1 (+) plasmid (100 ng), as described previously [32].

### 3.9. Isolation of Blood Cells and Hemolysis Assay

Blood from healthy volunteers was collected by venipuncture (Greiner Bio-One, Kremsmünster, Austria) into K2E K2EDTA VacuetteTM tubes (Greiner Bio-One, Kremsmünster, Austria) with 4 mL blood. The assay for hemolysis was adapted from Sæbo et al. [71]. Blood was centrifuged at 1000× *g* for 10 min, and plasma was kept for further use. Red blood cells were then resuspended in 10 mL PBS and centrifuged again at 1000× *g* for a total of three times. Cells were then counted using a hemocytometer and seeded at a cell number of 1 × 10^7^ cells/100 µL PBS per well in conical 96-well plates (ThermoFisher, Darmstadt, Germany). Next, 1:2 peptide dilutions starting from 40 µM either in PBS or 10% donor serum were prepared, and 100 µL was added to 100 µL of RBC. Plates were then incubated for one hour at 37 °C. As the positive control, three wells with 100 µL RBCs were treated with 100 µL PBS + 0.5% Triton X-100. After incubation, plates were centrifuged at 1000× *g* for 5 min and supernatants were transferred to flat 96-well plates. Optical density (OD) was determined at 450 nm using a GloMax^®^-Multi Detection System (Promega, Mannheim, Germany). Images from RBCs from light microscopy and centrifuged plates were taken using an iPhone 12 mini.

### 3.10. Isolation and Stimulation of PBMCs 

Isolation of PBMCs was performed by adding 3 mL blood on a 3 mL layer of Histopaque-1077 (Sigma Aldrich, Schnelldorf, Germany) and centrifuged at 400× *g* for 30 min at room temperature with the lowest brake and acceleration settings. After centrifugation, plasma was aspirated and the PBMC layer was transferred to a 15 mL conical centrifugation tube. Cells were resuspended in 10 mL PBS, followed by centrifugation at 250× *g* for 10 min. The cell pellet was resuspended in 5 mL PBS, followed by centrifugation at 250× *g* for 10 min for a total of three times. Finally, the pellet was resuspended in 1 mL cell culture medium supplemented with 10% FCS. Cells were then seeded in a 48-well plate at 2.5 × 10^5^ cells/125 µL per well, treated with 125 µL peptide dilutions in the absence and presence of 0.25 µg/mL lipopolysaccharide (LPS) from Escherichia coli (Sigma-Aldrich, Schnelldorf, Germany) and incubated at 37 °C for 24 h. Supernatants were then collected for subsequent analysis by ELISA. 

### 3.11. Enzyme-Linked Immunosorbent Assay (ELISA)

ELISA was performed using human tumor necrosis factor-α (TNF-α) and interleukin-6 (IL-6) ELISA MAX^TM^ De Luxe Sets from Biolegend (San Diego, CA, USA) according to the manufacturer’s instructions. Supernatants were diluted 1:5 for TNF-α and 1:500 for IL-6. 

### 3.12. SDS-PAGE and Western Blot Analysis

For determination of peptide stability, 20 µg peptide was added to 50 µL PBS or PBS with 10% FCS and incubated for 48 h at 37 °C or stored at −20 °C. For non-reducing conditions, 4 µL peptide solution was mixed with 1 µL H_2_O and 5 µL Novex™ Tricine SDS Sample Buffer (2×) (ThermoFisher, Darmstadt, Germany). For reducing conditions, 4 µL peptide solution was mixed with 1 µL NuPAGE™ Sample Reducing Agent (10×) (Invitrogen by Thermo Fisher Scientific) and 5 µL Novex™ Tricine SDS Sample Buffer (2×). Samples were then heated to 85 °C for 10 min. A total of 10 µL was then loaded onto each well of 10–20% Novex™ Tricine, 1.0 mm, Mini Protein Gels (Invitrogen/ThermoFisher, Waltham, MA, USA) and run in 1× Novex™ Tricine SDS Running Buffer (Invitrogen/ThermoFisher, Waltham, MA, USA) for 90 min at 100 V. After electrophoresis, gels were fixed with 40% methanol and 10% acetic acid followed by staining with 0.1% Coomassie brilliant blue R250 (Sigma-Aldrich, Schnelldorf, Germany) in 40% methanol and 10% acetic acid for one hour and destaining using fixing solution.

### 3.13. Molecular Modeling

MD simulations of TAT-I24 and various cysteine substitutions complexed to dsDNA (GC-hairpin oligonucleotide) were carried out as described previously [32]. The final 50 ns fractions of the trajectory were subjected to MM-PB(GB)SA analysis. Each system was considered independently 3 times, and statistical averages and variances were computed. Detailed analysis of complex forming residues was performed following the authors of [72]. Visualization of biomolecular structure was performed with VMD [73].

### 3.14. Data Analysis

Statistical analysis was performed using GraphPad Prism 8 (GraphPad Software, San Diego, CA, USA).

## 4. Conclusions

For potential use in antiviral therapy or prophylactic treatment of infections caused by dsDNA viruses, an antiviral peptide should be applicable systemically. Due to the potential disadvantages of synthetic peptides, such as aggregation, oligomerization or reduced serum stability, individual cysteine residues of TAT-I24 were replaced or deleted. While C14 of TAT-I24 could be replaced or deleted without a severe impact on its antiviral activity, the replacement of two and three cysteine residues caused reduced antiviral activity, depending on the virus model applied. The cyclization of the peptide between C14 and C20 had the most detrimental effect on antiviral activity. All tested peptides did not cause hemolysis at peptide concentrations < 20 µM and did not significantly alter the levels of pro-inflammatory cytokines IL-6 or TNF-α. Gel staining indicated oligomer formation in the solution of TAT-I24, while C14del showed a clearer monomeric peptide, indicating the beneficial effect of cysteine replacements on the physico-chemical properties of TAT-I24. These studies, therefore, serve as a basis for further steps in TAT-I24 engineering, analytics and development.

## 5. Patents

Hanna Harant is the inventor of patent application WO2019/057973 “gene expression inhibitors”.

## Figures and Tables

**Table 1:** Sensitivities to peptide variants of TAT-I24 in two infection models and DNA binding capacities of peptides. Amino-acid substitutions of TAT-I24 are indicated in bold red.

Name	Sequence	HEK293 IC_50_ [µM]	HEK293 CC_50_ [µM]	SI *	NIH/3T3 IC_50_ [µM]	DNA Binding IC_50_ [µM]
TAT-I24	GRKKRRQRRRPPQCLAFYACFC	0.05	124	2480	0.91	1.01
C14A	GRKKRRQRRRPPQ**A**LAFYACFC	0.08	67	838	0.92	2.11
C14L	GRKKRRQRRRPPQ**L**LAFYACFC	0.05	53	1060	0.57	2.19
C14G	GRKKRRQRRRPPQ**G**LAFYACFC	0.10	50	500	1.07	2.36
C14R	GRKKRRQRRRPPQ**R**LAFYACFC	0.10	106	1060	2.95	1.44
C14P	GRKKRRQRRRPPQ**P**LAFYACFC	0.09	92	1022	3.51	2.54
C14del	GRKKRRQRRRPPQ**-**LAFYACFC	0.10	37	370	0.92	1.26
C22G	GRKKRRQRRRPPQCLAFYACF**G**	0.05	71	1420	0.55	1.61
C22S	GRKKRRQRRRPPQCLAFYACF**S**	0.05	99	1980	1.19	1.35
C22del	GRKKRRQRRRPPQCLAFYACF**-**	0.08	n.d. **	n.d.	1.65	1.38
C22del_cyc ***	GRKKRRQRRRPPQCLAFYACF**-**	1.41	n.d.	n.d.	12.17	3.77
C20R, C22del	GRKKRRQRRRPPQCLAFYA**R**F**-**	0.05	66	1320	1.91	2.47
C14A, C20R, C22M	GRKKRRQRRRPPQ**A**LAFYA**R**F**M**	0.58	63	109	3.59	2.35
TAT	GRKKRRQRRRPPQ	[31]			[31]	

* SI—selectivity index; ** n.d.—not determined; *** Disulfide bond C14-C20; IC_50_ (half-maximal inhibitory concentration) and CC_50_ were calculated using non-linear regression (curve fit).

## Data Availability

The original contributions presented in the study are included in the article/Supplementary Material; further inquiries can be directed to the corresponding author.

## Supplementary Materials

The following supporting information can be downloaded at: https://www.mdpi.com/article/10.3390/ijms251910463/s1.
