# Supplementary material for: In Vitro Profiling of the Antiviral Peptide TAT-I24
Source: Int J Mol Sci. 2024 Sep 28;25(19):10463. doi: 10.3390/ijms251910463 (PMC11477294; doi:10.3390/ijms251910463)
Supplement: Supplementary file 1 [file ijms-25-10463-s001.zip › Figure S1_V2.0.pdf]

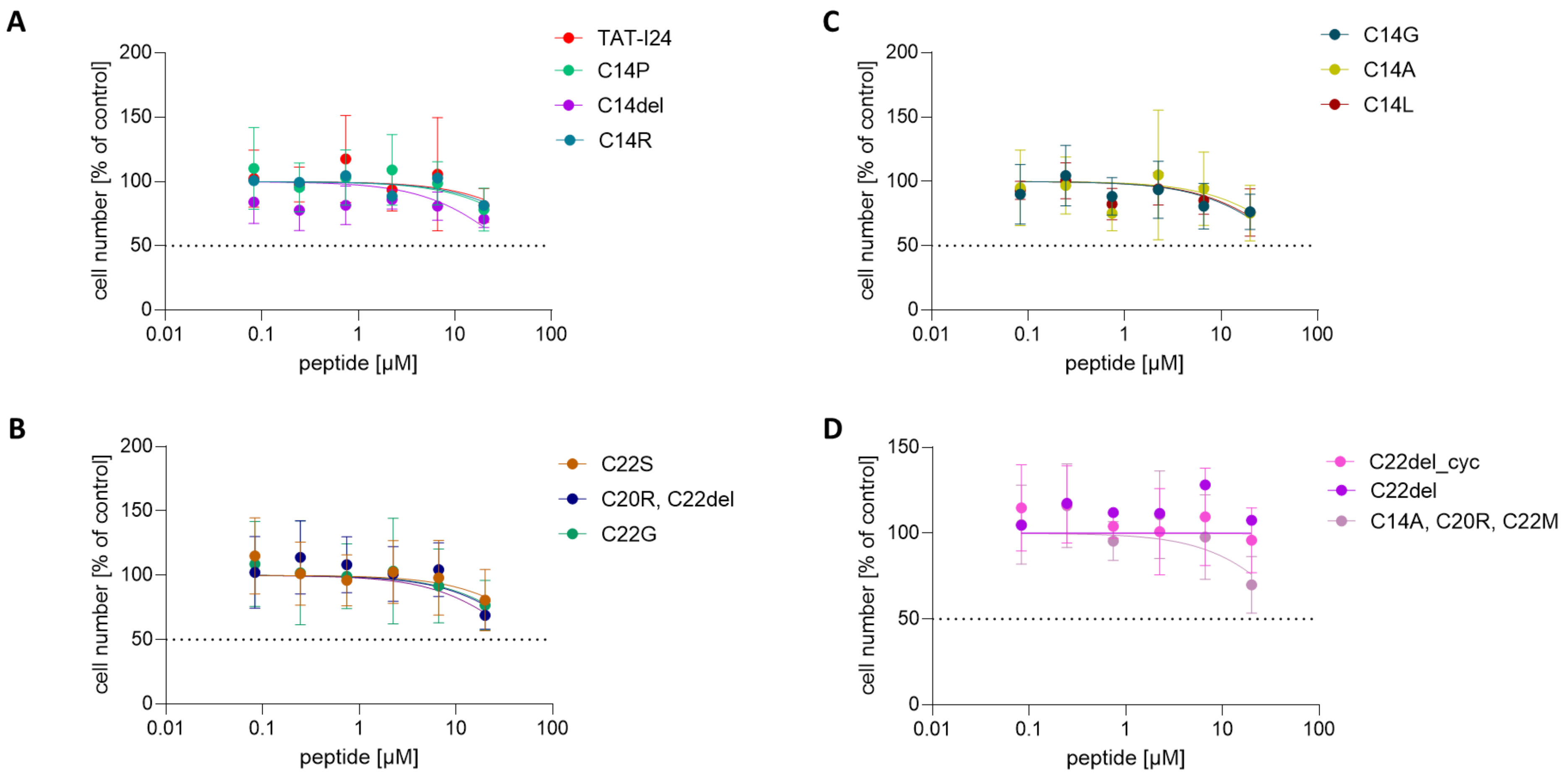

Figure S1. Cell viability assay in HEK293 cells in the presence of peptides. Cells were incubated with 3-fold dilution series of peptides starting with a concentration of 20 µM for 72 hours and measured using the Cell Titer Blue assay. Curve fitting of % viable cells of untreated control was performed using Graph Pad Prism software.
