# Supplementary material for: In Vitro Profiling of the Antiviral Peptide TAT-I24
Source: Int J Mol Sci. 2024 Sep 28;25(19):10463. doi: 10.3390/ijms251910463 (PMC11477294; doi:10.3390/ijms251910463)
Supplement: Supplementary file 1 [file ijms-25-10463-s001.zip › Figure S2.pdf]

Supplementary Figure 2

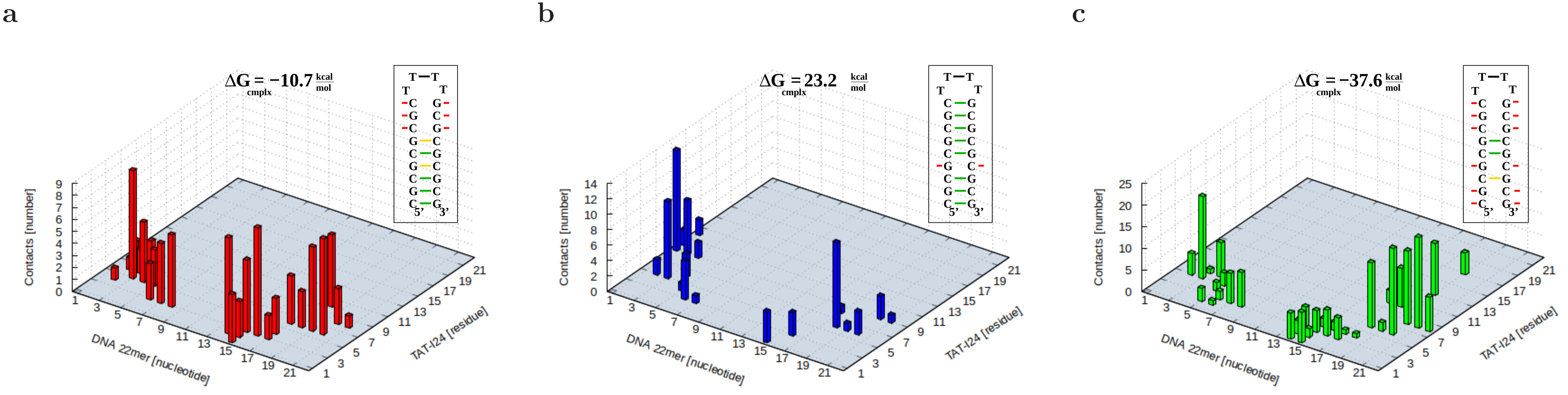

Complex forming pairs of TAT-I24 residues with DNA hairpin nucleotides from repeated MD simulations (a-c) in explicit water (AMBER-22, ff14SB, DNA.bsc1, TIP3P). a) Initial MD simulation (250 ns) followed by MM-PB(GB)SA analysis (restricted to the final 50 ns of the trajectory) [1] reveals a binding free energy of -10.7 kcal/mol together with a statistically dominant average structure of the complex. Subsequent analysis of native contacts in this average structure was carried out following [2]. Individual residues of the peptide TAT-I24 (GRKKRRQRRRPPQCLAFYACFC) are considered along the y-axis, while DNA nucleotides forming a helical hairpin are given on the x-axis with a schematic fold shown in the inset (regular base pairs: green, open bases: red, imperfect Watson-Crick base pairs: yellow). Complex forming pairs of amino acids with DNA nucleotides are shown as red columns at corresponding intersection points, where the height is a measure of the "strength" of the interaction, i.e. how many cross-contacts had been identified with distances smaller than 4 Å excluding hydrogen atoms. All 10 N-terminal TAT-I24 residues except R_9 are involved in complex formation where the interaction is mainly with the 5 base pairs starting at G_2-C_{21} up to and including the open bases C_7, G_8, G_{14}, C_{15} and G_{16} of the stem loop. **b)** Repeated MD simulation and analysis at otherwise identical conditions to a) with derived binding free energy of 23.2 kcal/mol, hence effectively disintegration free energy. Complex forming pairs are now given as blue columns and identify all 12 N-terminal TAT-I24 residues except R_2, Q_7 and P_{11} interacting with an array of 7 nucleotides on both strands of the hairpin. The latter maintains an almost perfect shape of only Watson-Crick base pairs barring G_4 and C_{19}. **c)** Repeated MD simulation and analysis identical to a) and b) yielding a binding free energy of -37.6 kcal/mol. Complex forming pairs are now given as green columns. The 6 N-terminal TAT-I24 residues together with R_8, R_{10}, Q_{13} and F_{17} are involved in complex formation to a large array of nucleotides on both strands of the stem loop. Many of the classic Watson-Crick base pairs have been broken up.

References

[1] H. Harant, S. Höfinger, F. Kricek, C. Ruf, Z. Ruzsics, H. Hengel, I.J.D. Lindley, "The Peptide TAT-I24 with Antiviral Activity against DNA Viruses Binds Double-Stranded DNA with High Affinity," *Biologics*, **1**, 41-60, (2021)

[2] `https://amberhub.chpc.utah.edu/detect-aminoacid-interactions`
