# Supplementary material for: In Vitro Profiling of the Antiviral Peptide TAT-I24
Source: Int J Mol Sci. 2024 Sep 28;25(19):10463. doi: 10.3390/ijms251910463 (PMC11477294; doi:10.3390/ijms251910463)
Supplement: Supplementary file 1 [file ijms-25-10463-s001.zip › Figure S3.pdf]

Supplementary Figure 3

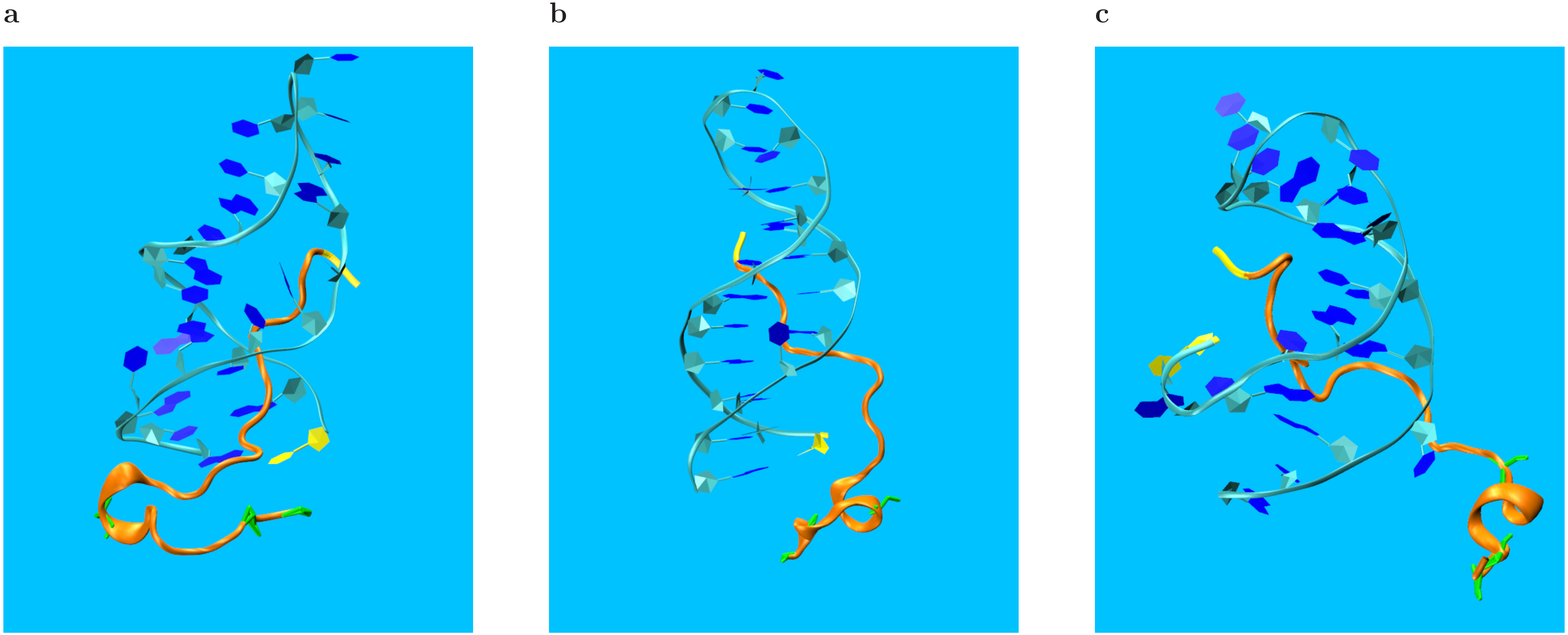

Average complex structures of TAT-I24 with DNA hairpins (made of C-G base pairs) as revealed from MM-PB(GB)SA analysis of 3 repeated MD simulations (a-c) in explicit water (AMBER-22, ff14SB, DNA.bsc1, TIP3P). a) TAT-I24 peptide (GRKKRRQRRRPPQCLAFYACFC) in cartoon representation (orange) with the N-terminus coloured in yellow forming a complex with the helical hairpin of the 22-nucleotide DNA given in ribbon representation (blue/cyan) where the 5'-end is also shown in yellow. All 3 CYS-residues at positions 14, 20 and 22 of the TAT-I24 peptide are represented as sticks coloured in green. Several of the Watson-Crick base pairs have broken up (see SI Fig 2a for a sketch of the helical hairpin and corresponding ΔG_{cmplx}). **b)** Similar representation to a) of the TAT-I24/DNA complex but for a repeated MD simulation carried out from scratch at otherwise identical conditions. Most of the Watson-Crick base pairs have maintained the classic conformation (also see SI Fig 2b). **c)** Similar representation to a) and b) of the TAT-I24/DNA complex but for another repeated MD simulation run again at identical conditions. The majority of the classic Watson-Crick base pairs has gone lost (also see SI Fig 2c).
