# Supplementary material for: In Vitro Profiling of the Antiviral Peptide TAT-I24
Source: Int J Mol Sci. 2024 Sep 28;25(19):10463. doi: 10.3390/ijms251910463 (PMC11477294; doi:10.3390/ijms251910463)
Supplement: Supplementary file 1 [file ijms-25-10463-s001.zip › SI Figure 4.pdf]

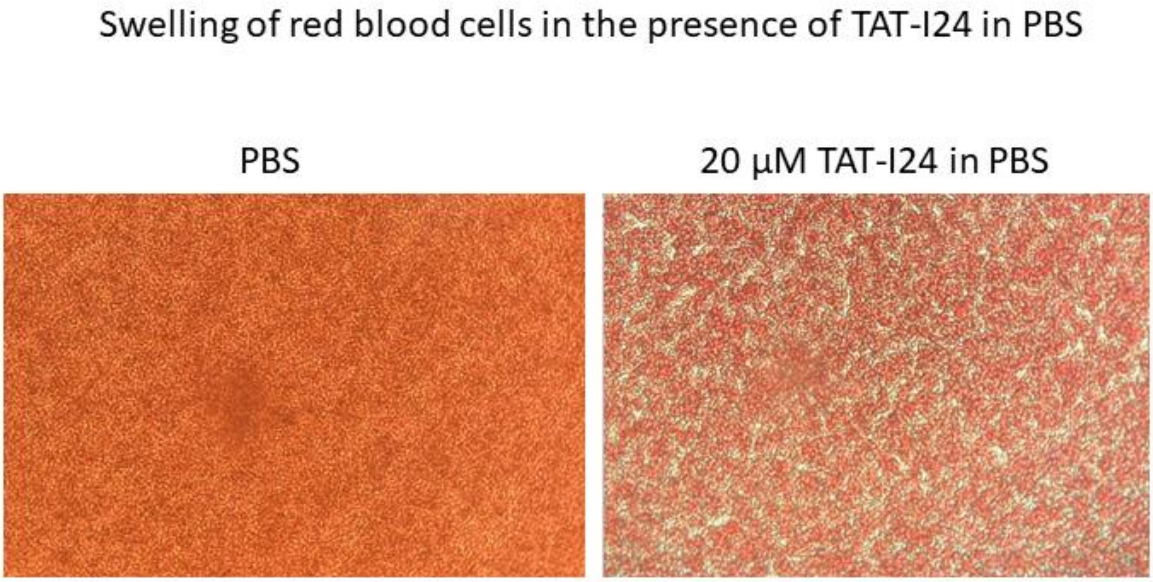

Figure S4. **Swelling of red blood cells in the presence of TAT-I24 in PBS.** Light microscopic image of red blood cells in PBS untreated or treated with 20 µM TAT-I24 in PBS for one hour.
